# Genetic Variability of *Polypedilum* (Diptera: Chironomidae) from Southwest Ecuador

**DOI:** 10.3390/insects13040382

**Published:** 2022-04-13

**Authors:** Isabel Ballesteros, Mishell Bravo-Castro, Santiago Villamarín-Cortez, Gabriela Jijón, Narcís Prat, Blanca Ríos-Touma, Christian Villamarín

**Affiliations:** 1 Grupo de investigación Biodiversidad, Medio Ambiente y Salud (BIOMAS), Facultad de Ingenierías y Ciencias Aplicadas (FICA), Universidad de Las Américas, Quito 170503, Ecuador; isabelballesteros@ucm.es (I.B.); anambravo@outlook.com (M.B.-C.); gabriela.jijon@udla.edu.ec (G.J.); blanca.rios@udla.edu.ec (B.R.-T.); 2 Departamento de Genética, Fisiología y Microbiología, Facultad de Biología, Universidad Complutense de Madrid, 28040 Madrid, Spain; 3 Department of Biology, Program in Ecology, Evolution and Conservation Biology, University of Nevada, Reno, NV 89557, USA; sanbiol@gmail.com; 4 Instituto Nacional de Biodiversidad–INABIO, Rumipamba 341 y Av. Shyris, Quito 170135, Ecuador; 5 Grupo de Investigación Freshwater, Hydrology and Ecology Management (FHEM), Departamento de Ecología, Universidad de Barcelona, 08014 Barcelona, Spain; nprat@ub.edu

**Keywords:** diversity, Andean biogeography, Chironomidae, *Polypedilum*

## Abstract

**Simple Summary:**

*Polypedilum* is a genus of aquatic non-biting midges in the family Chironomidae. This genus is widely distributed in neotropical rivers from lowlands to Andean highlands. Nevertheless, making species identification based on morphology is quite complex, even more so in the Neotropics, since systematic studies of this group are scarce. DNA barcoding can help to overcome this problem using a short DNA sequence as a barcode for species delimitation. A fragment of the mitochondrial gene cytochrome c oxidase I (CO1) has been successfully employed as a barcode in the genus *Polypedilum.* In this study, our aim was to understand the effect of environmental characteristics on *Polypedilum* diversity and distribution. We examined the CO1 sequence of 68 *Polypedilum* specimens from rivers with different environmental conditions located in an important biogeographic area of Ecuador. We identified five morphotypes and seven putative species which revealed high genetic variability among them. *Polypedilum* distribution seems to be affected mainly by two environmental factors, dissolved oxygen, and temperature. Our study is the first evidence of richness within the genus in Ecuador, highlighting the importance of developing taxonomic studies along with ecological assessments to further describe and identify new species.

**Abstract:**

Chironomids show a wide distribution and can occupy several habitats due to their high adaptive capacity in different freshwater environments. The genus *Polypedilum* is found along a wide elevational and environmental gradient in the neotropics, and its genetic variability could help to elucidate factors determining its distribution and tolerance to the environmental changes of different species or populations. This study examines the genetic variability of *Polypedilum* in an important biogeographic area that acts as a geographical barrier of biodiversity at the border of the Choco and Tumbes biomes. We identified five *Polypedilum* morphotypes using classic taxonomic methods. We examined 68 *Polypedilum* individuals from eight sampling sites in El Oro Province, Ecuador, analyzing the putative molecular species using the cytochrome c oxidase subunit 1 (CO1) mitochondrial gene fragment. Then, we calculated molecular diversity indices, Haplotype diversity (Hd), and θs and θπ estimators. Seven *Polypedilum* OTUs were determined from which a high molecular diversity was registered. A CCA was conducted to understand the population composition in relation to environmental characteristics. Results indicated that dissolved oxygen and temperature are the main environmental factors affecting *Polypedilum* distribution across elevational gradients and between basins.

## 1. Introduction

Chironomids are vastly distributed around the world, having developed high resistance and resilience to diverse environmental stressors [1]. They are considered pioneer organisms that colonize aquatic ecosystems after natural or anthropogenic stress periods [2]. Natural disturbances such as hydrologic and discharge intensity changes, seasonality, and habitat heterogeneity can affect the distribution of chironomid community assemblages [3,4]. However, environmental quality is highlighted as one of the most important factors influencing chironomid composition and diversity [3,5]. Additionally, other biotic factors in combination with geographical isolation, physical barriers, and distinct environmental conditions can influence changes in riverine populations, limiting their dispersal capacity [6,7].

Elevation, out of all the physical barriers, has been emphasized as a determinant factor in chironomid community composition changes [5]. For instance, some chironomid clades such as Orthocladiinae, Podonominae and Diamesinae are dominant in highland Andean rivers, while others, like the Chironominae and Tanypodinae clades, are more predominant in lowland rivers [3,5,8]. However, genus distribution is also related to additional factors like environmental conditions, geographical location, and life history. *Barbadocladius* (Chironomidae, Orthocladiinae)*,* mainly distributed in coldwater rivers in Central and Southern Andes, has been studied across a latitudinal gradient in the Andes [9]. These studies highlighted the importance of elevation as a geographic barrier, along with temperature and distance as important features. Nonetheless, several genera are widely distributed throughout an elevational range, such as *Cricopotus*, *Parametriocnemus*, *Chironomus*, *Rheotanytarsus*, *Polypedilum.* These genera’s distribution is determined by a high adaptation capacity or tolerance to environmental changes across elevation and local environmental characteristics, but it remains unclear which environmental filters act as distribution drivers at a species-level.

*Polypedilum* is vastly distributed worldwide and includes more than 500 described species [10]. It is mostly distributed in neotropical rivers from the lowlands [5] to Andean highlands [3,8]. *Polypedilum* tolerates moderately polluted waters, making it a biological indicator of environments with moderate organic matter concentrations and reduced dissolved oxygen levels [11,12]. However, there is a taxonomic and ecological knowledge gap for this genus. Factors such as taxonomic complexity and a high number of cryptic species can make genus-level identification difficult at larval stages [10,13]. Also, in neotropical species where chironomid systematics are not well developed, it is even more difficult to address ecological questions at species level.

To overcome species-level morphological identification problems, DNA barcoding has been extensively applied as a tool in taxonomy and ecology to study biodiversity and make species-level identifications feasible [14,15]. In this study, we used taxonomic and molecular characteristics, and environmental factors, to understand *Polypedilum* distribution and responses to environmental variations. Different approaches were used for DNA barcode sequence analysis, creating genetic clusters known as operational taxonomic units (OTUs) and putative species [16,17]. Distance-based approaches such as the Automatic Barcode Gap Discovery (ABGD) [18], and tree- based approaches like the multi-rate Poisson tree process (mPTP) [19] are commonly used in barcoding studies and have been useful in *Polypedilum* species identification [13,15].

This study pioneers the use of DNA barcoding in tandem with different environmental conditions to examine *Polypedilum* diversity, never used before in any other Chironomid study in Ecuador, or in the ecology of El Oro Province, Ecuador. Our study site was located at the beginning of the Huancabamba depression, which is an important biogeographical barrier between Northern and Central Andes. We examined putative molecular species using the cytochrome c oxidase subunit 1 (CO1) mitochondrial gene fragment as the barcoding region. Our aim was to understand distribution drivers of *Polypedilum* OTUs across an elevational gradient in El Oro Province. We hypothesized that a local environmental characteristic, like temperature, can explain genetic variability. On the other hand, distance can act as a biogeographical barrier for all species in this study, because in general Chironomids do not fly long distances. Therefore, distance can limit the distribution ranges to either high or low elevation zones and basins, and we hypothesized that genetic difference would increase as distance increases.

## 2. Materials and Methods

### 2.1. Study Area and Specimen Collection

**Figure 1:**
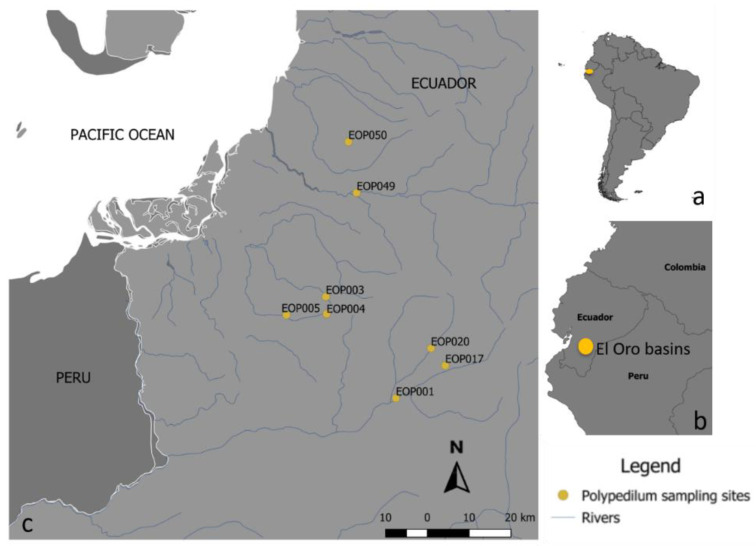
Study sites: (**a**) sites in relation to South America; (**b**) site distribution in Ecuador; and (**c**) study sites in El Oro Province. B1: Arenillas basin (EOP003, EOP004, EOP005); B2: Puyango basin (EOP001, EOP017, EOP020); B3: Jubones basin (EOP049); B4: Siete river basin (EOP050).

The present study was conducted at eight sampling sites located in El Oro Province in southwestern Ecuador. The site number is based on three elevational bands: three sites are located below 500 masl (EOP049, EOP050 and EOP005); two sites are between 501 and 1000 m.a.s.l. (EOP020 and EOP001); and three sites are located above 1000 m.a.s.l. (EOP003, EOP004, EOP017). These sites are within four different basins: Arenillas (B1), Puyango (B2), Jubones (B3) and Siete River (B4) (Figure 1, site codes are included in the figure legend). These basins are characterized by three vegetation types: montane forest, foothill forest and lowland dry forest [5].

### 2.2. Field and Laboratory Procedures

Several physicochemical variables were measured on site, including dissolved oxygen (DO), pH, conductivity, total dissolved solids (TDS), and temperature. We used a multiparameter HACH 5465060 (Loveland, CO, USA) to measure these environmental variables. We collected nine Surber samples (0.11 m^2^ each, 1 m^2^ total per site) per site using a 280 microns mesh size. Samples were fixed in 96% alcohol for DNA preservation.

We identified Chironomids to genus-level and separated them into morphotypes. For this, heads were slide-mounted using the Epler’s method [20], and these preparations were used to confirm morphological identification using specialized bibliography for the *Polypedilum* genus [20,21,22,23,24]. The remaining sample fragments were preserved in 96% alcohol for genetic analysis. Only third and fourth instar larvae were taxonomically determined to be able to include all the taxonomical characteristics (HC: Head capsule; AS: Antennal segment; M: Mentum teeth length; PP: Paralabial plates) and minimize missleading identifications by larval developmental maturity and variability. The taxonomical characteristics were identified and measured usin a microscope Olympus BX51 (Olympus, PA, USA) connected to a digital camera (Lumera, ON, Canada). Nevertheless, DNA identification and molecular anlayses were conducted for all individuals.

### 2.3. DNA Extraction and PCR Amplification

Micropestles were used to homogenize the tissue, and DNA extraction was carried out, following the CTAB protocol (Hexadecyltrimethylammonium bromide) with a Phenol-chloroform purification [25]. DNA was eluted in 40 µL, and DNA quantity and quality were measured with a NanoDrop2000 (Thermo Scientific™, Waltham, MA, USA). Then, electrophoresis was performed. An initial PCR was performed using 2 μL of DNA and the LCO1490/HCO2198 (0.2 µM) primers [26] with a total volume of 15 μL. An annealing temperature of 45 °C was used and 45 amplification cycles were performed on this reaction. Then, a nested PCR was carried out using as template 5 μL of a 1:100 dilution of the initial PCR and the MT6/NANCY primers (0.2 µM) [27]. An annealing temperature of 45 °C was used, and 32 amplification cycles were performed. PCR amplification was confirmed by agarose electrophoresis and directly sequenced at UDLA research laboratory.

DNA isolation and PCR analysis were performed for at least 15 specimens per sampling site. Nevertheless, the number of sequences obtained was lower. Sequences were edited and aligned with MEGA X software [28,29,30]. Sequences were translated into amino acids to check for the absence of insertions, deletions or stop codons. Sequence were deposited in GenBank under accession number MW021054–MW021121. The code sequences indicate river basin, sampling station and specimen number.

### 2.4. Species Delimitation and Phylogenetic Reconstruction

To delimitate potential species (operational taxonomic units: OTUs) within the currently recognized *Polypedilum* larvae, we used two different approaches: Automatic Barcode Gap Discovery (ABGD) method and multi-rate Poisson tree process (mPTP). We added mtCOI *Polypedilum* sequences from Chile (BOLD:ACY5605; ACY5280; ACY5279; ACY5313; ACY6050) and Colombia (MN366023–MN366028) to the available alignment. The ABGD method clustered sequences into hypothetical species named OTU (Operational Taxonomic Unit). This grouping was based on differences between intra and interspecific distance variation [18]. We applied the K2P and JC69 models with a relative width of one and maximum *p* value (the prior maximum divergence of intraspecific diversity) of 0.25, using the online ABGD server (https://bioinfo.mnhn.fr/abi/public/abgd/, accessed on 6 August 2021). The multi-rate Poisson tree process (mPTP) is a phylogeny-aware approach [19]. The PTP analysis used a rooted phylogenetic input tree constructed with raxmlGUI version 2.0, using 500 replicates and the GTR + G + I nucleotide substitution model [31]. The mPTP analysis was implemented into the webserver (http://mptp.h-its.org/, accessed on 6 August 2021), using a multi-rate Poisson tree process model and following default settings.

In addition to species identification, phylogenetic relationships were examined using the MEGA X software [30] and a Maximum likelihood inference with Tamura-Nei model according to the Akaike Information Criterion (AIC). *Barbadocladius* sp. (KF386118.1) was used as outgroup.

Pairwise distances were calculated under a Kimura-2 parameter model [32] in MEGA X. Arlequin 3.5 [33] was used to calculate molecular diversity indices, Haplotype diversity (Hd), and θs and θπ estimators.

### 2.5. Environmental Data Analysis of El Oro Rivers as Drivers of Polypedilum Assemblage Structure

To address the environmental variability within the studied sites, we used the measured physicochemical parameters in a Principal Component Analysis (PCA). First, we conducted a correlation analysis between variables, to find a correlation between elevation and temperature and between conductivity and TDS values. Elevation and TDS were removed from the analysis because of their collinearity with temperature and conductivity, respectively. This analysis reduced the dimensionality of multiple variables measured in rivers and allowed for pattern understanding among environmental variables [34]. Additionally, we overlapped a cluster analysis with a cluster of Euclidean distances similarity to show site groupings based on environmental variability. These analyses were carried out using Primer 6 software [35]. A Canonical Correspondence Analysis (CCA) using CANOCO (4.5, Microcomputer Power, Ithaca, NY, USA) was performed to explore the influence of environmental characteristics on *Polypedilum* OTUs assemblages [36]. This analysis arranges the ecological optimum along canonical axes using the physicochemical variables [37].

## 3. Results

### 3.1. Environmental Characteristics of El Oro Watersheds

All rivers in El Oro province have shown high environmental variability, especially in conductivity measurements, showing a high variability in the Arenillas river basin ($\bar{x}$ = 99.45 μS/cm) and a very low in the Siete river basin (31 μS/cm). There was a low pH and temperature variation among sites. The Arenillas river had the highest pH ($\bar{x}$ = 7.72) specially in EOP004 (Table 1). The dissolved oxygen (DO) content was similar among all sites except for EOP017, which presented the lowest value (Table 1).

Our PCA showed that main variables explaining the differences among sites for the X axis (52.6% of variance explained) were elevation (0.501), dissolved oxygen (−0.576) and temperature (−0.544), and for the Y axis (33.3% of variance explained) were pH (−0.685) and conductivity (−0.572). The two axes explained 85.8% of the accumulated variation.

**Figure 2:**
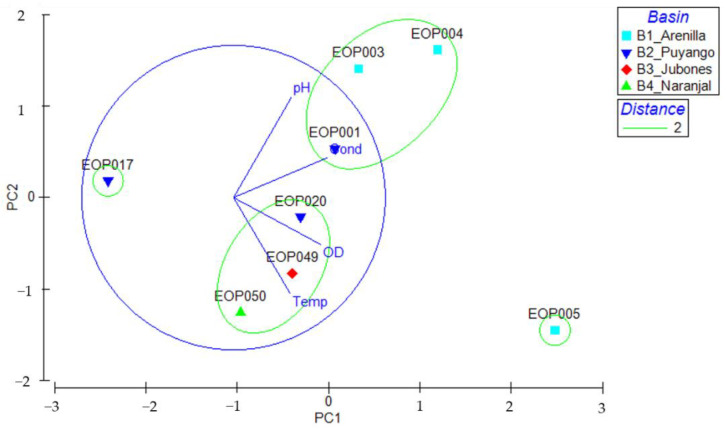
Principal Component Analysis (PCA) for environmental variables with overlaid average clustering from El Oro Province watersheds: B1: Arenillas, B2: Puyango, B3: Jubones, and B4: Siete River.

The cluster analysis clearly defined two groups in relation to the environmental characteristics in the study sites. The first group included the EOP001, EOP003 and EOP004 sites, two sampling sites located above 1000 m.a.s.l. and one at 529 m. The second group consisted of EOP049, EOP050 and EOP020 were between 0 and 1000 m.a.s.l. (Figure 2), showing a strong response to temperature (and elevation). It is important to point out that the rivers that make up each group are geographically close. Two sites did not group with other localities, the first site showed a high DO concentration, the second site showed low DO and conductivity values.

### 3.2. El Oro Polypedilum Morphotypes

**Figure 3:**
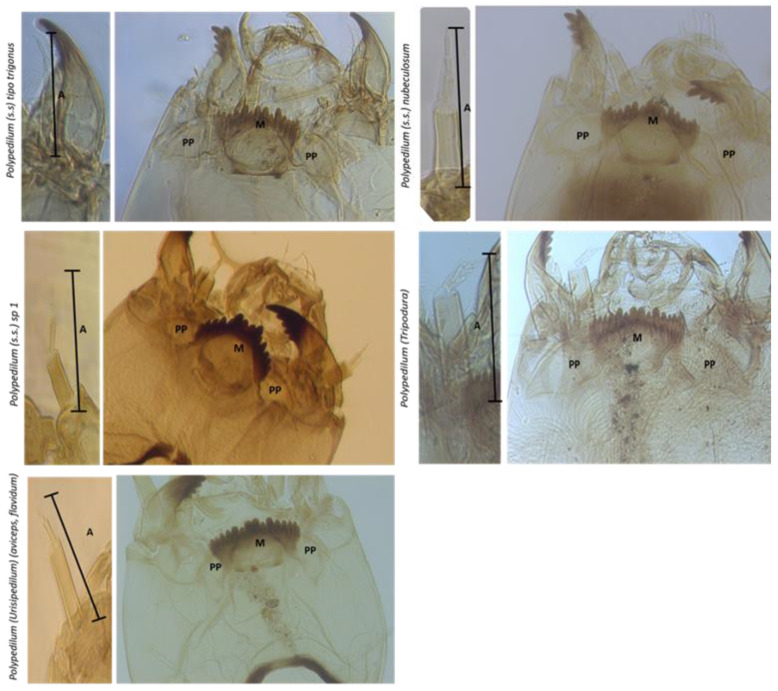
*Polypedilum* morphotypes in El Oro. A: Antenna; M: Mentum; PP: Paralabial plates.

The morphological analysis clearly defined five *Polypedilum* morphotypes in El Oro sites. The characteristics that differentiated each morphotype were antennal ratio, mentum teeth configuration and paralabial plates (Table 2). The *Polypedilum* (*s.s.*) *gr trigonus* morphotype has a unique mentum tooth configuration with a shorter fourth tooth in relation to the third and fifth teeth (Table 2, Figure 3). In contrast, *Polypedilum* (*s.s.*) *nubeculosum* and *Polypedilum* (*Tripodura*) have a downward tooth configuration from the third to the fifth tooth (Table 2, Figure 3). The distinguishing characteristic between *P.* (*s.s.*) *nubeculosum* and *P.* (*Tripodura*) is in antennal length. Another discriminating characteristic is that *P.* (*s.s.*) *nubeculosum* possesses a dark spot under the mentum (Figure 3). *Polypedilum* (*s.s.*) *sp1* show central teeth below the first mentum teeth (Figure 3). *Polypedilum* (*Urisipedilum*) shows paralabial plates with extended posterior lobes (Figure 3).

### 3.3. Species Delimitation and Phylogenetic Analysis

We obtained a 474-bp-long fragment from the mtCO1 gene from 68 *Polypedilum* sp. specimens in eight sampling stations in El Oro Province (Figure 1). Sequence comparisons against databases indicated an identity match of 85% to 90% with previously entered *Polypedilum* sequences. Most of the closest matches in the databases belong to specimens unclassified at species level. We also included nine *Polypedilum* sp sequences for outgroup comparisons. Two *Polypedilum* sequences are from Llanquihue and Petruhue in Chile and seven are from Colombia.

**Figure 4:**
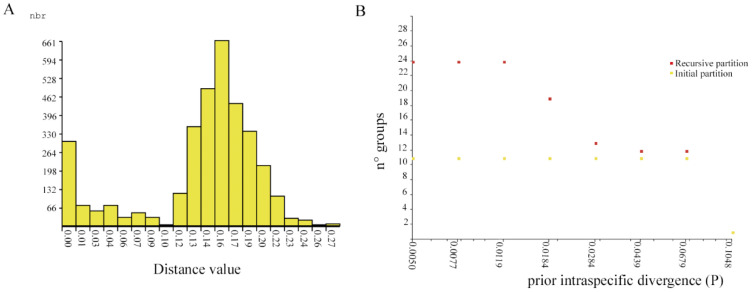
Results generated from ABGD method: (**A**) pairwise Jukes Cantor distances histogram. The horizontal axis shows the pairwise JC-distance, and the vertical axis shows the number of pairwise sequence comparisons; (**B**) number of OTUs with prior intraspecific divergence based on DNA barcodes of *Polypedilum* specimens from Ecuador, Colombia and Chile.

The ABGD method produced the same results for the JC69 and K2P models. The frequency histogram of pairwise distances indicated a clear barcode gap between 0.01 and 0.12 (Figure 4A). This method produced one initial partition and 12 OTUs (*p* = 0.01 to 0.077), while the recursive partition generated from 25 (*p* = 0.005) to 13 (*p* = 0.044) OTUs (Figure 4B). According to the initial partition results, specimens from El Oro were grouped into seven OTUs that could be assigned to putative species. Sequences from three sampling stations located in the Chinchiná river basin in Colombia made up one species [38] and a single OTU. Sequences from the two sites in Chile were grouped into two OTUs, while recursive partitioning analysis (*p* = 0.044) yielded three separate groups. However, in the BOLD platform they were divided into four different species. Thus, DNA barcode clusters exhibited exclusive distributions, indicating some geographical influence in grouping.

**Figure 5:**
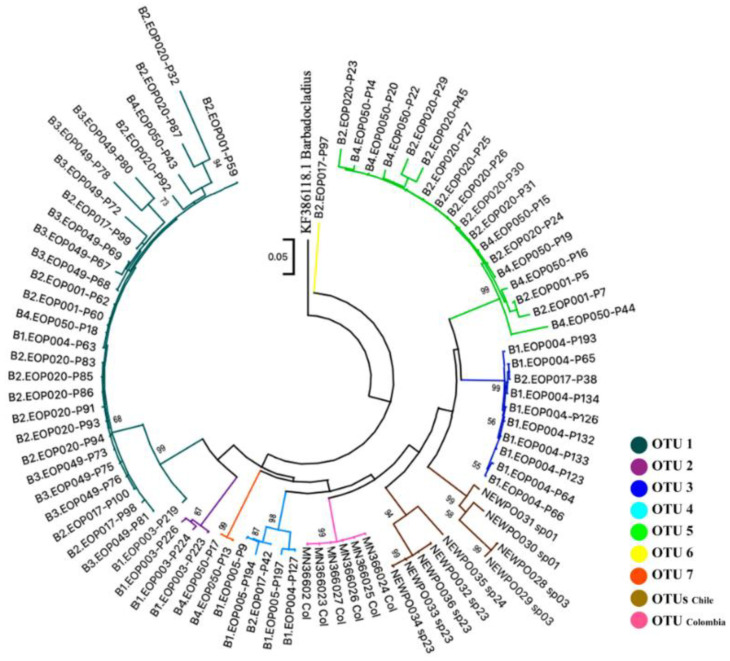
Maximum likelihood tree of *Polypedilum* specimens based on partial COI sequences and the Tamura-Nei model. *Barbadocladius* sp. appears as an outgroup. Branch numbers are 500 replicate bootstrap support (>50%); scaling represents K2P genetic distance; distinct colors represent different OTUs.

The phylogenetic analysis supported the groups inferred from the ABGD method. The mPTP model produced a total of 11 clusters. In the mPTP (Figure S1) and ML analyses (Figure 5), the specimens grouped in each OTU formed a well-supported monophyletic clade except for specimen p32 that was clustered in the OTU1 clade. Nevertheless, some distant geographical populations formed nested clades. For example, Llanquihue’s (Chile) OTU with El Oro’s OTUs 3 and 5 were grouped in the same clade. DNA barcode clusters from Chile showed a high genetic dissimilarity among specimens. Chilean specimens were divided into four species in the BOLD database.

Nevertheless, there were some inconsistencies between the taxonomic study and the OTUs defined in the phylogenetic analysis of *Polypedilum* of El Oro. The specimens assigned to *Polypedilum (s.s.) gr trigonus* and *P. (Tripudura)* belong to different OTUs, and each one showed inconsistencies between the two approaches of identification. While the sequences of the specimens assigned to the rest of morphotypes are grouped in the same OTU, *P. nubeculosum* is grouped in OTU 1 with *P. sp 1* and *P.* (*Urisipedillum*) sequences. Even some individuals assigned to sp1 and *P.* (*Urisipedillum*) would correspond to different putative species (OTU) according to DNA barcoding analysis. The pairwise distance within sequences of the same *Polypedilum* morphotype ranged from 0.007 (*P. (Tripudura))* to 0.11 *(P.* (*Urisipedillum*)), while that of *Polypedilum (s.s.) sp1* and *Polypedilum (Urisipedilum)* showed the highest distances (Table 2).

**Figure 6:**
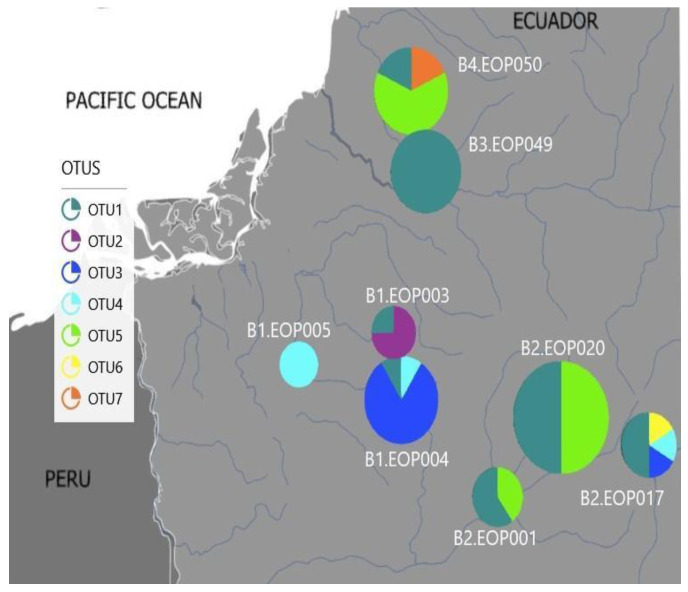
El Oro’s putative species geographical distribution in El Oro Province. B1: Arenillas river basin, B2: Puyango river basin, B3: Jubones river basin, and B4: Siete River river basin.

We considered seven OTUs from El Oro that had an intraspecific divergence ranging from 0.4% to 5% for further analysis (Table 3). These OTUs had a dissimilarity ranging from 17% to 30%. These results demonstrated a high *Polypedilum* species diversity (based on the number of OTUs) in El Oro Province. The species separation analysis indicated three abundant *Polypedilum* putative species that included OTUs 1, 3 and 5. Specimens from four river basins in El Oro were included in OTU1, making it the most abundant and widespread OTU. Specimens from Arrenillas and Puyango basins were grouped into OTU3 and OTU4, respectively, while individuals from the Puyango and Siete basins were included in OTU5. OTU2 and 7 had only one to three individuals collected from a single river basin (Figure 6). It is important to highlight that EOP003, EOP004 and EOP017 sites are located between 1015 and 1293 masl, while the other sites are located below 991 m.a.s.l.

### 3.4. Environmental Characteristics and Polypedilum OTUs Population Composition

**Figure 7:**
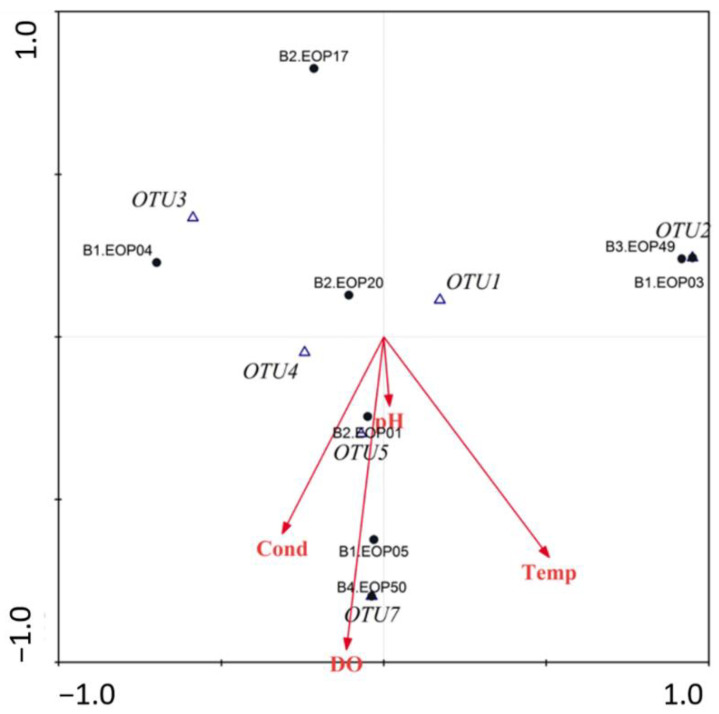
Canonical correspondence analysis of environmental variables and *Polypedilum* OTU abundance at el Oro Province, Ecuador (Elev: elevation, Cond: conductivity, pH: hydrogen potential, DO: dissolved oxygen): possible species determined with the ABGD analysis. B1: Arenillas river basin, B2: Puyango river basin, B3: Jubones river basin, and B4: Siete River river basin.

Individuals from OTU 1, 2 and 3 were found at higher elevations, except for the EOP049 site specimens, that were found at a lower altitude (grouped in OUT 1). In contrast, individuals from OTU 5 and 7 were all found at a lower elevation, high DO concentrations and warmer water temperatures (Figure 7). Individuals from the EOP005 locality were grouped into OTU4 and their grouping appeared to not be influenced by any of the environmental variables measured. The OTU6 group, with only one specimen, was not used in the analysis (Figure 7).

### 3.5. Polypedilum Genus Diversity Indices

We identified 228 polymorphic sites and 56 haplotypes with an overall gene diversity (Hd) of 0.998 and nucleotide diversity of 0.122. All populations were genetically diverse with a nucleotide diversity between 0.042 and 0.123 (Table 4). Specimens from the EP049 site showed the lowest nucleotide diversity and molecular diversity estimator values among all El Oro sites. In contrast, specimens from the EP017 site had the highest molecular diversity index values and lowest number of sequenced individuals.

## 4. Discussion

Molecular data can especially help in the species identification of immature specimens from poorly known groups and specimens with underdeveloped, morphologically important characteristics [39]. Several studies [13,15,40] have pointed out the need and utility of *Polypedilum* species determination, given the prevalence of cryptic species within the genus. As with many other studies [10,23,41], we found that DNA barcoding identification did not agree with morphological identification, as seen in genera like *Cryptochironomus*, *Kiefferulus* and *Polypedilum*. In our study, some morphotypes of *Polypedilum* were not grouped into a corresponding OTU. It is important to consider that morphological identifications are complicated by underdeveloped characteristics in immature larvae. Even though the sampling design included 15 *Polypedilum* individuals at each site, not all the larvae were in the third or fourth instar. As a result, we could not compare the morphotype identification to the OTU assignment in the molecular analysis. This highlights the importance of developing taxonomic studies that include larval, pupal, and adult stages. Nevertheless, taxonomic work on larvae might show limitations to identify at species level so the use of morphotype or morphotaxa in these initial instars, supported by DNA barcoding analysis, might be the best way [42]. Thus, taxonomic studies are especially important for groups that are not well known as chironomids.

However, molecular differences seemed to be linked to altitude. In the Andes, altitude increase is mostly related to temperature decrease. Another important ecological factor was DO. A decrease in DO concentrations could be related to pollution. However, oxygen concentrations can change throughout the day and year, and may not exert as much influence on genetic variability as temperature. Although the influence of altitude on aquatic communities [43] and Chironomids [3] is well-known, it is important to understand the impact of altitude on little-known groups. Elevation may have a greater impact on organisms that are more widely distributed than *Polypedilum.* However, it is important to consider the influence of altitude on the genetic variability and putative species in future ecology and taxonomic works.

Other factors besides elevation may be important, including genetic drift and a short life cycle. Genetic drift can play an important role in speciation, especially if the adult dispersion is difficult due to geographical barriers [44]. Therefore, *Polypedilum* adults, like other aquatic macroinvertebrates [44], can produce up to 24 generations per year, which may be possible due to high temperatures and abundant trophic resources in this area.

Our results suggest that larval morphotypes may be influenced by local environmental characteristics and genetic drift. *Polypedilum* larvae are also very similar and it is difficult to make species-level identifications. In general, pupal exuviae and adults may provide more information than larvae. Additionally, DNA barcoding is needed to make accurate species identifications, especially in areas with scarce taxonomic information.

The ABGD and mPTP methodologies yielded similar results. The ABGD analysis identified eight putative species in El Oro sampling stations while the tree-based approach (mPTP) identified seven. Differences in molecular species determination could be due to collection size and the Chironomids instar limitation, and it is expected that a higher sample number would reveal more cryptic species in El Oro. We considered the phylogeny-based method, i.e., the mPTP method, a more accurate method for species identification. This method takes into account evolutionary relationships to barcode match without depending on threshold settings [19]. For some Dipteran species the threshold value of 4% was set based on previous studies [15,45,46]. The intraspecific differences found within larval morphotypes are greater than those found within OTUs. These differences are not equal to those previously estimated for *Polypedilum*. Song et al. (2018) determined a maximum intraspecific divergence of 10.8% for *Polypedilum masudai* (Tokunaga) and reported large intraspecific divergences in cryptic species

In the Neotropics, environmental variability (climate and temperature) tends to be more stable than in higher latitudes [47]; however, in the Andes a variety of environmental factors that change with elevation become important diversity drivers [5,48]. The Andes are comprised of narrow tropical environmental gradients, isolating organisms by their ecological plasticity [5,47]. Small elevational changes in the Andes result in big environmental changes, which influence diversification of cryptic species [49]. Our results suggest that there is a high diversity level within the *Polypedilum* genus, and it contains a high number of cryptic species.

Therefore, environmental characteristics in El Oro Province act as drivers of chironomid community diversity. In this case, the Tumbesine dry climate, the Choco humid Bioregion, and the Huancabamba depression act as geographical barriers and drive speciation [5,9,43]. Additionally, some studies suggest that elevation could act as a principal driver of high chironomid genus-level richness [3,5,8]. The *Polypedilum* genus is widely distributed from Southern to Northern Ecuador from highland streams and rivers to those at sea level. This genus diversity shows an association with environmental changes across an elevational gradient, where drivers such as local environment and natural history produce high species richness at lower elevations.

## 5. Conclusions

*Polypedilum* is a complex taxonomic genus influenced by local environmental factors, such as elevation and dispersal capacity within and between basins. The adaptation process to warmer areas makes *Polypedilum* very diverse in low altitude areas due to the high environmental variability, and results in a high number of cryptic species. This group, as well as other little-studied groups, should be prioritized in research and conservation programs. Thus, this study shows the taxonomy and molecular complexity of the genus. *Polypedilum* larvae in advanced instar stages have distinctive taxonomic characteristics, allowing for morphospecies separation. However, initial larval stages are difficult to classify, making molecular analysis interpretation more challenging.

These groups are valuable due to their functional and adaptive capacity. This group’s richness is underestimated, like that of other aquatic macroinvertebrate taxa. It is very important to develop taxonomic studies to further describe and identify new species, mainly of the less studied groups like chironomids. Our study clearly pointed out that molecular tools are fundamental in characterizing the real diversity of this group. These complementary studies (morphological and molecular) are of major importance, particularly in areas with unique biogeographic characteristics as our study area.

## Figures and Tables

**Table 1:** Environmental characteristics of the studied sites.

Code	Basin	pH	Dissolved Oxygen (mg/L)	Conductivity (μS/cm)	Temperature (°C)	Altitude (m.a.s.l.)
EOP003	Arenilla	7.8	11.69	92.6	20.8	1260
EOP004	Arenilla	8.2	14.69	92.6	20.8	1015
EOP005	Arenilla	7.16	18.76	113.2	24.3	252
EOP001	Puyango	7.2	12.69	92.6	20.8	529
EOP017	Puyango	6.75	8.76	31.8	20	1293
EOP020	Puyango	7.37	10.98	58.1	22.8	991
EOP049	Jubones	7.1	11.52	53.4	23.2	121
EOP050	Siete	6.66	14.61	31	21.7	205

**Table 2:** El Oro’s Polypedilum morphotypes measurements, mean (min-max). HC: Head capsule; AS: Antennal segment; MC: Mentum central teeth length; M1: Mentum first teeth length; PP: Paralabial plates. p-dist: Pairwise distance.

Taxa	HC Width (min/max)	AS2/AS3-5	LS3/AS3	MC/M1	M4/M3-5	PP-lóbpost	PP Wid/Length	PP W/Intp	p-Dist
*Polypedilum* (*s.s.*) *gr trigonus*	320.88 (309.79–349.82)	0.54 (0.43–0.72)	>>	2.19 (1.98–2.67)	3 > 4 < 5	No	2.28 (2.04–2.47)	2.14 (2.00–2.26)	0.0272952
*Polypedilum* (*s.s.*) *nubeculosum*	313.93 (240.41–388.73)	0.50 (0.43–0.67)	>>	1.43 (0.85–2.06)	3 > 4 > 5	No	2.40 (2.05–3.19)	2.30 (2.00–2.79)	0.0588167
*Polypedilum* (*s.s.*) *sp1*	287.73 (301.20–550.14)	0.88 (0.80–0.98)	>>	0.44 (0.34–0.53)	3 > 4 > 5	No	2.29 (1.77–2.64)	2.45 (1.89–3.16)	0.0963929
*Polypedilum* (*Tripodura*)	209.24 (176.79–240.28)	0.82 (0.67–1.04)	<<	1.79 (1.47–2.08)	3 > 4 > 5	No	2.49 (2.32–2.60)	4.69 (3.97–5.13)	0.0067183
*Polypedilum* (*Urisipedilum*)	329.80 (299.09–380.44)	1.26 (1.13–1.34)	>>	1.76 (1.52–1.90)	3 > 4 > 5	Si	1.44 (1.28–1.58)	1.55 (1.48–1.64	0.1136894
*Polypedilum* sp Colombia									0
*Polypedilum* sp3 Chile									0
*Polypedilum* sp1 Chile									0.0382833
*Polypedilum* sp23 Chile									0.0393383

**Table 3:** Mean distances calculated using Kimura 2 parameter model within each OTU and morphotype. N: number of specimens in each group; S.E: standard error. n/c: not calculated.

OTUs	N	Mean Distance	S.E.
OTU1	29	0.048	0.005
OTU2	3	0.015	0.005
OTU3	10	0.006	0.002
OTU4	5	0.043	0.008
OTU5	18	0.029	0.004
OTU6	1	n/c	n/c
OTU7	2	0.0042	0.003

**Table 4:** *Polypedilum* genus genetic diversity from eight sampling sites in El Oro Province stations.

Sampling Site	Number of Sequences	Number of Segregating Site (S)	Number of Haplotype (h)	Haplotype Diversity (Hd)	Nucleotide Diversity (Pi)	Theta_S	s.d. Theta_S	Theta_pi	s.d. Theta_pi
EOP001	5	78	4	0.900	0.095	37.440	18.967	45.200	27.780
EOP003	4	64	4	1.000	0.070	34.909	19.100	33.167	22.067
EOP004	12	113	9	0.945	0.050	32.093	12.918	23.818	12.815
EOP005	3	32	3	1.000	0.046	21.333	13.103	21.667	16.591
EOP017	6	115	6	1.000	0.123	50.364	23.942	58.400	34.119
EOP020	18	141	14	0.961	0.113	40.994	14.469	53.673	27.235
EOP049	10	73	9	0.978	0.042	25.804	10.728	19.800	10.837
EOP050	10	138	10	1.000	0.115	48.781	19.960	54.622	29.235

## Data Availability

Sequences obtained in this work are available at the GenBank database through the NCBI Entrez retrieval system (https://www.ncbi.nlm.nih.gov/, accessed on 1 April 2022). Accession numbers: MW021054–MW021121.

## Supplementary Materials

The following are available online at https://www.mdpi.com/article/10.3390/insects13040382/s1, Figure S1: mPTP species delimitation output tree. Red lines indicate the groups considered as a single species.

## References

[1] Ashe P., Murray D.A., Reiss F. (1987). The zoogeographical distribution of Chironomidae (Insecta: Diptera). Annls Limnol..

[2] Lencioni V., Cranston P., Makarchenko E.A. (2018). Recent advances in the study of Chironomidae: An overview. J. Limnol..

[3] Acosta R., Prat N. (2010). Chironomid assemblages in high altitude streams of the Andean region of Peru. Fundam. Appl. Limnol..

[4] Koperski P. (2019). Phylogenetic diversity of larval Chironomidae (Diptera) in lowland rivers as a potential tool in assessment of environmental quality. Hydrobiologia.

[5] Villamarín C., Villamarín-Cortez S., Salcido D.M., Herrera-Madrid M., Ríos-Touma B. (2021). Drivers of diversity and altitudinal distribution of chironomids (Diptera: Chironomidae) in the Ecuadorian Andes. Rev. Biol. Trop..

[6] Miller M.P., Blinn D.W., Keim P. (2002). Correlations between observed dispersal capabilities and patterns of genetic differentiation in populations of four aquatic insect species from the Arizona White Mountains, U.S.A. Freshw. Biol..

[7] Finn D.S., Encalada A.C., Hampel H. (2016). Genetic isolation among mountains but not between stream types in a tropical high-altitude mayfly. Freshw. Biol..

[8] Hamerlik L., Da Silva F.L., Jacobsen D. (2018). Chironomidae (Insecta: Diptera) of Ecuadorian highaltitude streams: A survey and illustrated key. Fla. Èntomol..

[9] Prat N., Ribera C., Rieradevall M., Villamarín C., Acosta R. (2013). Distribution, abundance and molecular analysis of genus *Barbadocladius* Cranston & Krosch (Diptera, Chironomidae) in tropical, high altitude Andean streams and rivers. Neotrop. Èntomol..

[10] Cranston P., Martin J., Spies M. (2016). Cryptic species in the nuisance midge *Polypedilum nubifer* (Skuse) (Diptera: Chironomidae) and the status of *Tripedilum* Kieffer. Zootaxa.

[11] Cranston P., Armitage P.D., Cranston P.S., Pinder L.C. (1995). Introduction. The Chironomidae: Biology and Ecology of Non-Biting Midges.

[12] Luoto T.P. (2011). The relationship between water quality and chironomid distribution in Finland—A new assemblage-based tool for assessments of long-term nutrient dynamics. Ecol. Indic..

[13] Song C., Wang Q., Zhang R., Sun B., Wang X. (2016). Exploring the utility of DNA barcoding in species delimitation of *Polypedilum* (*Tripodura*) non-biting midges (Diptera: Chironomidae). Zootaxa.

[14] Previšić A., Gelemanović A., Urbanič G., Ternjej I. (2016). Cryptic diversity in the Western Balkan endemic copepod: Four species in one?. Mol. Phylogenetics Evol..

[15] Song C., Lin X.-L., Wang Q., Wang X.-H. (2018). DNA barcodes successfully delimit morphospecies in a superdiverse insect genus. Zool. Scr..

[16] Hebert P.D.N., Cywinska A., Ball S.L., Dewaard J.R. (2003). Biological identifications through DNA barcodes. Proc. R. Soc. B Biol. Sci..

[17] Hebert P.D.N., Ratnasingham S., De Waard J.R. (2003). Barcoding animal life: Cytochrome c oxidase subunit 1 divergences among closely related species. Proc. R. Soc. B Boil. Sci..

[18] Puillandre N., Lambert A., Brouillet S., Achaz G. (2012). ABGD, Automatic Barcode Gap Discovery for primary species delimitation. Mol. Ecol..

[19] Kapli P., Lutteropp S., Zhang J., Kobert K., Pavlidis P., Stamatakis A., Flouri T. (2017). Multi-rate Poisson tree processes for single-locus species delimitation under maximum likelihood and Markov chain Monte Carlo. Bioinformatics.

[20] Epler J. (2001). Identification manual for the larval Chironomidae (Diptera) of North and South Carolina. A Guide to the Taxonomy of the Midges of the Southeastern United States, including Florida.

[21] Prat N., Rieradevall M., Acosta R., Villamarín C. Guía para el Reconocimiento de las Larvas de Chironomidae (Diptera) de los Ríos Altoandinos de Ecuador y Perú. Clave para la Determinación de los Géneros; 2011; p. 78. http://www.ub.edu/riosandes/docs/CLAVE_LARVAS_PERU_ECUADORvfoto3_v7.pdf.

[22] Epler J., Ekrem T., Cranston P. (2013). The larvae of Chironominae (Diptera: Chironomidae) of the Holarctic region—Keys and diagnoses. The Larvae of Chironomidae of the Holarctic Region—Keys and Diagnoses.

[23] Saether O.A., Andersen T., Pinho L.C., Mendes H.F. (2010). The problems with *Polypedilum* Kieffer (Diptera: Chironomidae), with the description of *Probolum* subgen. n. Zootaxa.

[24] Bolton M.J. (2012). Ohio EPA Supplemental Keys to the Larval Chironomidae (Diptera) of Ohio and Ohio Chironomidae Checklist.

[25] Cerda-Granados D., Díaz V. (2013). Optimización de un protocolo de extracción de ADN genómico para *Pinus tecunumanii*. Encuentro.

[26] Folmer O., Black M., Hoeh W., Lutz R., Vrijenhoek R. (1994). DNA primers for amplification of mitochondrial cytochrome c oxidase subunit I from diverse metazoan invertebrates. Mol. Mar. Biol. Biotechnol..

[27] Simon C., Frati F., Beckenbach A., Crespi B., Liu H., Flook P. (1994). Evolution, weighting, and phylogenetic utility of mitochondrial gene sequences and a compilation of conserved polymerase chain reaction primers. Annals Èntomol. Soc. Am..

[28] Altschul S.F., Gish W., Miller W., Myers E.W., Lipman D.J. (1990). Basic local alignment search tool. J. Mol. Biol..

[29] Ratnasingham S., Hebert P.D.N. (2007). BOLD: The barcode of life data system: Barcoding. Mol. Ecol. Notes.

[30] Kumar S., Stecher G., Li M., Knyaz C., Tamura K. (2018). MEGA X: Molecular evolutionary genetics analysis across computing platforms. Mol. Biol. Evol..

[31] Silvestro D., Michalak I. (2011). RaxmlGUI: A graphical front-end for RAxML. Org. Divers. Evol..

[32] Kimura M. (1980). A simple method for estimating evolutionary rates of base substitutions through comparative studies of nucleotide sequences. J. Mol. Evol..

[33] Excoffier L., Lischer H.E.L. (2010). Arlequin suite ver 3.5: A new series of programs to perform population genetics analyses under Linux and Windows. Mol. Ecol. Resour..

[34] Jolliffe I.T., Cadima J. (2016). Principal component analysis: A review and recent developments. Philos. Trans. R. Soc. A Math. Phys. Eng. Sci..

[35] Clarke K., Gorley R. (2006). PRIMER v6: User Manual/Tutorial (Plymouth Routines in Multivariate Ecological Research). PRIMER-E..

[36] Braak Ter C., Barendregt P. (1998). CANOCO. Reference and Manual User’s Guide to Canoco for Windows: Software for Canonical Community Ordination (Version 4).

[37] Borcard D., Gillet F., Legendre P. (2011). Numerical Ecology with R.

[38] Grueso-Gilaberth R.N., Jaramillo-Timarán K.S., Ospina-Pérez E.M., Richardi V.S., Ossa-López P.A., Rivera-Páez F.A. (2020). Histological description and histopathology in *Polypedilum* sp. (Diptera: Chironomidae): A potential biomarker for the impact of mining on tributaries. Annals Èntomol. Soc. Am..

[39] Geraci C.J., Al-Saffar M.A., Zhou X. (2011). DNA barcoding facilitates description of unknown faunas: A case study on Trichoptera in the headwaters of the Tigris River, Iraq. J. N. Am. Benthol. Soc..

[40] Carew M., Pettigrove V., Hoffmann A.A. (2003). Identifying chironomids (Diptera: Chironomidae) for biological monitoring with PCR–RFLP. Bull. Èntomol. Res..

[41] Song C., Wang X., Bu W., Qi X. (2020). Morphology lies: A case-in-point with a new non-biting midge species from Oriental China (Diptera, Chironomidae). ZooKeys.

[42] Rossaro B., Marziali L., Montagna M., Magoga G., Zaupa S., Boggero A. (2022). Factors controlling morphotaxa distributions of diptera Chironomidae in freshwaters. Water.

[43] Villamarín C., Rieradevall M., Prat N. (2020). Macroinvertebrate diversity patterns in tropical highland Andean rivers. Limnetica.

[44] González-Trujillo J.D., Petsch D.K., Córdoba-Ariza G., Rincón-Palau K., Donato-Rondon J.C., Castro-Rebolledo M.I., Sabater S. (2019). Upstream refugia and dispersal ability may override benthic-community responses to high-Andean streams deforestation. Biodivers. Conserv..

[45] Lin X., Stur E., Ekrem T. (2015). Exploring genetic divergence in a species-rich insect genus using 2790 DNA barcodes. PLoS ONE.

[46] Nzelu C.O., Cáceres A.G., Arrunátegui-Jiménez M.J., Lañas-Rosas M.F., Yañez-Trujillano H.H., Luna-Caipo D.V., Holguín-Mauricci C.E., Katakura K., Hashiguchi Y., Kato H. (2015). DNA barcoding for identification of sand fly species (Diptera: Psychodidae) from leishmaniasis-endemic areas of Peru. Acta Trop..

[47] Gaston K.J. (2000). Global patterns in biodiversity. Nature.

[48] Callisto M., Linares M.S., Kiffer W.P., Hughes R.M., Moretti M.S., Macedo D.R., Solar R. (2021). Beta diversity of aquatic macroinvertebrate assemblages associated with leaf patches in neotropical montane streams. Ecol. Evol..

[49] Dos Santos D.A., Molineri C., Nieto C., Zuñiga M.C., Emmerich D., Fierro P., Pessacq P., Rios-Touma B., Márquez J., Gomez D. (2018). Cold/Warm stenothermic freshwater macroinvertebrates along altitudinal and latitudinal gradients in Western South America: A modern approach to an old hypothesis with updated data. J. Biogeogr..

