# Supplementary material for: Genetic Variability of Polypedilum (Diptera: Chironomidae) from Southwest Ecuador
Source: Insects. 2022 Apr 13;13(4):382. doi: 10.3390/insects13040382 (PMC9028585; doi:10.3390/insects13040382)
Supplement: Supplementary file 1 [file insects-13-00382-s001.zip › insects-1672321-supplementary.pdf]

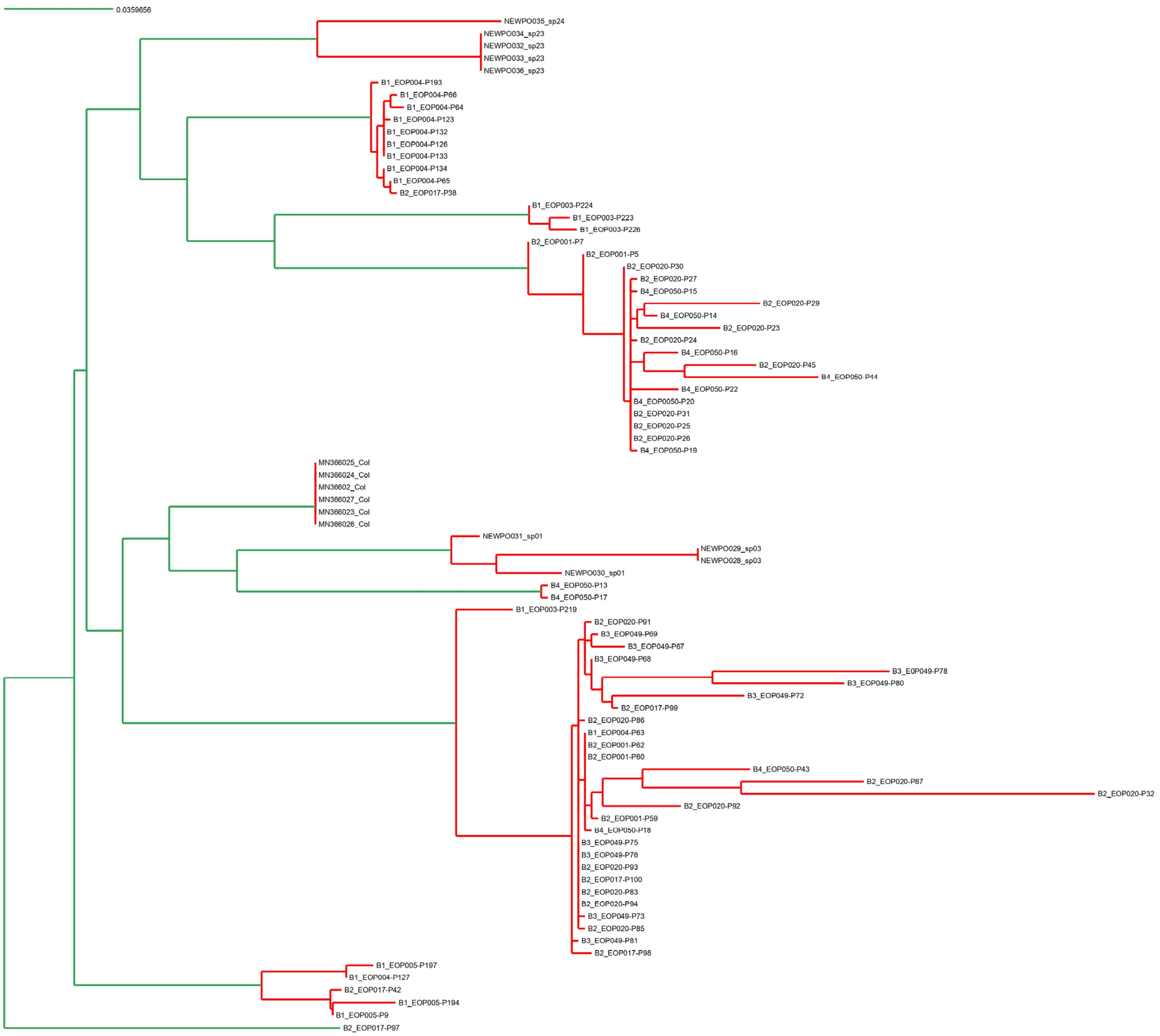

Figure S1: mPTP species delimitation output tree. Red lines indicate the groups considered as a single species.
